# Chronic kidney disease–mineral and bone disorder as multidimensional bone–vascular risk framework: from biochemical control to risk-and context-guided care

**DOI:** 10.3389/fendo.2026.1954515

**Published:** 2026-10-29

**Authors:** Guanting Chen, Tao Li, Kang Yang, Yaoxian Wang, Linqi Zhang

**Affiliations:** 1 Department of Nephrology, First Affiliated Hospital of Henan University of Chinese Medicine, Zhengzhou, Henan, China; 2 Collaborative Innovation Center of Prevention and Treatment of Major Diseases by Chinese and Western Medicine, Zhengzhou, Henan, China; 3 Henan University of Chinese Medicine, Zhengzhou, Henan, China

**Keywords:** bone-vascular axis, CKD-MBD, FFGF23–Klotho axis, renal osteodystrophy, secondary hyperparathyroidism, vascular calcification

## Abstract

Chronic kidney disease-mineral and bone disorder (CKD-MBD) is a systemic complication that spans early CKD, dialysis and kidney transplantation. It is better understood not as an isolated disturbance of calcium and phosphate metabolism but as a multidimensional, dynamic syndrome in which mineral-endocrine dysregulation, skeletal remodeling, skeletal fragility and structural consequences, and vascular calcification interact to shape patient outcomes. This narrative review argues that management should move beyond the control of biochemical targets toward phenotype-informed, evidence-graded and outcome-oriented care organized around the bone–vascular axis. We first examine why surrogate-based management has under performed and then propose a working multidimensional framework based on four interrelated domains mineral and endocrine status, bone turnover and mineralization, skeletal fragility and structural consequences, vascular calcification and cardiovascular risk, and clinical modifiers and treatment context. We also grade the evidence behind the principal mechanisms and interventions, from phosphate retention, the FGF23-Klotho axis, vitamin D-PTH dysregulation, Wnt/β-catenin signaling and calciprotein particles to inflammation, gut dysbiosis and uremic toxins, distinguishing mechanistic plausibility from observational association, surrogate-endpoint trials and hard-endpoint benefit. We then match diagnostic tools, including bone turnover markers, DXA, HR-pQCT, 18F-NaF PET/CT, vascular calcification scoring, calcification propensity testing and bone biopsy, to the clinical decisions they are meant to inform, and reframe treatment of hyperphosphatemia, secondary hyperparathyroidism, CKD-associated osteoporosis and vascular calcification by multidimensional framework. The recurring theme is that biochemical improvement is not itself benefit: the field needs stratified, testable strategies anchored in fracture, cardiovascular events, hospitalization, mortality, quality of life and physical function.

## 1 Introduction: why conventional CKD-MBD management remains inadequate

Chronic kidney disease (CKD) is a major component of the global public health burden. Global Burden of Disease analyses put the number of people living with CKD in the hundreds of millions worldwide and link the disease closely to cardiovascular mortality, progression to kidney failure and heavy cost of health care (1–3). CKD-MBD differs from many other complications in one important respect: it does not wait for the uremic stage. Endocrine compensation and bone-vascular remodeling begin early, as the glomerular filtration rate falls. For years a patient may show only mildly abnormal serum phosphate, parathyroid hormone (PTH), alkaline phosphatase or 25-hydroxyvitamin D, yet these seemingly biochemical changes eventually translate into fracture, vascular and valvular calcification, heart failure, hospitalization and death (4–7). Thus, CKD-MBD is less an electrolyte problem than a window onto the systemic reach of CKD.

Early KDIGO guideline reserved the term renal osteodystrophy (ROD) for bone pathology confirmed by histomorphometry, and defined CKD-MBD more broadly as a systemic disorder of mineral and bone metabolism caused by CKD, expressed through biochemical abnormalities, disordered bone turnover, mineralization, mass or strength, and vascular or soft-tissue calcification, alone or in combination (8–10). Placing skeletal disease and cardiovascular calcification within one disease spectrum encouraged clinicians to read serum phosphate, calcium and PTH not as isolated numbers but as risk signals for distant skeletal and vascular events.

Accumulating clinical evidence and experience have highlighted the limitations of this model and provide the rationale for the present review. Management has traditionally been organized around biochemical surrogate endpoints, although serum phosphate, calcium, and PTH do not show simple or consistent relationships with fracture, cardiovascular events, or mortality (4–7, 10). PTH reflects the mineral-endocrine and parathyroid milieu rather than directly measuring bone turnover; thus, a phosphate/PTH-predominant profile may suggest, but does not establish, high-turnover bone disease. Interpretation requires consideration of CKD stage, treatment exposure, and clinical context, as biochemical changes may not parallel skeletal turnover. Because reduced bone mineral density can result from diverse disorders, skeletal fragility should be assessed independently of turnover. Turnover assessment should integrate serial PTH, bone-specific alkaline phosphatase, and other markers; when results are discordant and treatment decisions depend on accurate classification, bone biopsy with histomorphometry remains the reference standard. Limited trial evidence in advanced CKD and dialysis necessitates balancing fracture risk, therapeutic uncertainty, and potential safety concerns (11–17). Vascular calcification represents a separate, potentially coexisting risk domain and is influenced by more than calcium and phosphate burden, including inflammation, oxidative stress, gut dysbiosis, calciprotein particles (CPPs), and phenotypic switching of vascular smooth muscle cells (18–30).

This review is therefore built around a single argument: CKD–MBD care should move beyond the pursuit of biochemical targets toward multidimensional, evidence-graded, and outcome-oriented bone–vascular risk management. Three commitments follow from this premise. First, rather than restating the syndrome as a set of mutually exclusive phenotypes, we propose in Section 2 an orthogonal framework comprising mineral–endocrine status; bone turnover and mineralization; bone mass, architecture, and fracture risk; vascular calcification and cardiovascular risk; and relevant clinical modifiers and treatment contexts. These domains may coexist within the same patient and are not intended to imply a validated classification or a single dominant phenotype. Second, for each major mechanism and intervention, we distinguish mechanistic plausibility from observational associations, surrogate-endpoint effects, and evidence for hard clinical outcomes, with this grading summarized in **Table 1**. Third, we present diagnostic tools and treatments in relation to specific clinical decisions rather than as inventories. Throughout, we emphasize that biochemical improvement alone does not constitute patient benefit unless it translates into improved clinical outcomes.

**Table 1:** Evidence for key CKD-MBD therapeutic targets and interventions.

Mechanism/intervention	Mechanistic plausibility	Observational association	RCT surrogate-endpoint evidence	Hard-endpoint (fracture/CV/mortality) evidence	Clinical readiness
Phosphate lowering (binders)	High	Consistent	Yes (lowers phosphate)	Absent/inconsistent; no mortality RCT (49)	Standard of care, target-guided
Calcimimetics (etelcalcetide, cinacalcet)	High	Yes	Yes (lower PTH) (63, 64)	Neutral (EVOLVE: no significant ITT reduction in death/CV) (69)	Established for SHPT, not for hard outcomes
Active vitamin D/analogues	High	Mixed	Yes (lower PTH) (68)	Uncertain; hypercalcemia risk	Selective (severe progressive SHPT)
Parathyroidectomy	High	Cohort/meta; survival signal (67)	Limited (no large RCT)	Observational only	Reserved for refractory/nodular SHPT
Denosumab	High	Yes (BMD)	Yes (BMD in selected CKD/transplant cohorts) (14, 82)	No dedicated advanced-CKD fracture RCT; hypocalcemia harm signal (14, 15, 115)	Use with strict monitoring
Romosozumab	High	Emerging	Yes (BMD, vertebral fracture in mild–moderate CKD) ADDIN NE.Ref.{EC9A5976-B5F1-46D1-A145-95586211C990} (13, 82, 83)	Absent in advanced CKD; CV safety unsettled	Investigational in advanced CKD
Teriparatide	Moderate	Small prospective (116, 117)	Limited	Absent	Selective (low-turnover/low BMD)
SNF472	High	—	Phase 2b: slows calcification (29)	Not yet (events/survival unproven)	Investigational
Vitamin K	Moderate	Mixed	Inconsistent RCTs (125–127)	Absent	Investigational
Magnesium	Moderate	Observational (87)	Limited	Absent	Investigational
Gut microbiota/anti-inflammatory/senescence targeting	Emerging	Preclinical/small human	Sparse	Absent	Experimental

### 1.1 Search strategy and selection criteria

We searched PubMed, Embase and Web of Science for English-language articles published up to early 2026, combining the terms “CKD-MBD”, “ROD”, “vascular calcification”, “secondary hyperparathyroidism”, “FGF23”, “Klotho”, “CPPs”, “bone turnover” and “fracture” with “chronic kidney disease”, “dialysis” and “kidney transplantation”. We gave priority to KDIGO guidelines and controversies-conference reports, randomized controlled trials, systematic reviews and meta-analyses, large observational cohorts, and representative mechanistic studies, and we favored more recent and higher-quality evidence where sources conflicted. Because this is a narrative rather than a systematic review, selection was interpretive and aimed at building the argument above rather than at exhaustive coverage; readers seeking pooled effect estimates are referred to the cited systematic reviews and meta-analyses. The review focuses on adults; pediatric CKD-MBD, which involves growth and skeletal maturation and is supported by a partly distinct evidence base, is beyond its scope.

## 2 Conceptual shift: from “renal osteodystrophy” to a multidimensional bone-vascular framework

For decades, ROD served as the principal histopathological framework for describing CKD-related bone disease. Defined by bone biopsy and histomorphometry, it characterized skeletal lesions according to turnover, mineralization, and bone volume, and historically included patterns such as bone turnover status, osteomalacia, mixed uremic osteodystrophy, and adynamic bone disease (8, 31, 32). Its major strength was its pathological resolution, providing a tissue-level description of skeletal lesions within the turnover–mineralization–volume framework. However, ROD is invasive and not readily repeatable, and it does not capture all determinants of skeletal fragility, including bone mineral density, microarchitecture, prior fracture history, and clinical risk factors, or extra-skeletal manifestations of CKD-MBD, such as vascular and valvular calcification, myocardial hypertrophy, and soft-tissue calcification. ROD should therefore remain a tissue-level descriptor of turnover, mineralization, and volume, integrated into a multidimensional framework in which mineral and endocrine abnormalities, skeletal fragility, cardiovascular and calcification risk, and clinical modifiers are assessed as coexisting rather than mutually exclusive domains.

In the KDIGO definition, the syndrome is a systemic disorder of mineral and bone metabolism caused by CKD that can appear across three domains: abnormal biochemistry, including calcium, phosphate, PTH, vitamin D and FGF23; abnormal bone turnover, mineralization, volume, linear growth and strength; and vascular or other soft-tissue calcification (8–10). This shared vocabulary made cross-disciplinary work possible and helped explain why fracture risk and cardiovascular risk so often climb together in CKD.

The 2025 KDIGO CKD-MBD Controversies Conference took stock of this progress. Its published conclusions, which are a controversies-conference report rather than a guideline update, proposed that future work give greater weight to two clinical syndromes, CKD-associated osteoporosis and CKD-associated cardiovascular disease, while judging that the 2017 guideline remains broadly consistent with current evidence (33). The reorientation toward clinical syndromes is useful because markers such as PTH and serum phosphate flag risk but do not answer the questions a patient actually asks: am I likely to fracture, do I need anti-osteoporotic treatment, will my vascular calcification progress, and does it raise my risk of cardiovascular death.

At the center of any such account is the bone-vascular axis. Bone is not only a mineral reservoir but an endocrine organ that helps regulate phosphate, FGF23, PTH and vitamin D, while the vessel wall, in the uremic milieu, can adopt an osteoblast-like phenotype and form calcified foci (20–24, 34). Factors released or regulated by bone act on the vasculature, and the inflammatory state of the vessel wall and immune system feeds back onto bone remodeling. Fracture risk and vascular calcification risk therefore overlap in CKD, though they are not driven by an identical mechanism.

The bone-vascular axis is a mechanistic framework rather than a validated clinical decision tool. To facilitate its clinical application, we organize the available evidence into five recurring, partially overlapping working phenotypes rather than treating CKD-MBD as a single undifferentiated syndrome. These categories are defined by characteristic patterns of clinical and biochemical findings and by differing diagnostic and therapeutic priorities (**Table 2**): a high-phosphate/high-PTH pattern generally associated with high bone turnover; a low-turnover pattern that may be encountered in the context of diabetes, older age, or sustained calcium loading; a calcification-dominant pattern characterized by established vascular or valvular calcification; an inflammation–malnutrition pattern in which systemic inflammation, protein–energy wasting, and, potentially, gut dysbiosis are prominent; and a post-transplant pattern shaped by persistent hyperparathyroidism, glucocorticoid exposure, and hypophosphatemia. These phenotypes are intended as provisional, educational categories rather than validated diagnostic classes or prescriptive management algorithms. They may coexist within the same patient, and their prospective validation remains an important research priority (Section 8). This proposed multidimensional framework is intended to complement, rather than replace, the 2025 KDIGO two-syndrome model of CKD-associated osteoporosis and CKD-associated cardiovascular disease. It serves as an organizing framework: the mechanisms discussed in Section 4, as well as the diagnostic tools and treatments reviewed in Sections 5 and 6.

**Table 2:** Multidimensional bone-vascular framework in CKD-MBD.

Framework	Dominant features	Key diagnostic combination	Treatment emphasis	Main outcome at stake
mineral and endocrine status	High phosphate, high PTH, raised bone-specific alkaline phosphatase, bone pain	PTH trend, bone-specific alkaline phosphatase, phosphate, imaging where needed	Phosphate control, calcimimetics or active vitamin D, parathyroidectomy if refractory	Fracture, calcification
bone turnover and mineralization	Low or normal PTH, diabetes, older age, high calcium load	Low PTH, bone-specific alkaline phosphatase, DXA/HR-pQCT, bone biopsy in complex cases	Reduce calcium and vitamin D load, avoid over-suppression, caution with antiresorptives	Fracture, calcification
Vascular calcification and cardiovascular risk	Established vascular or valvular calcification, high calcium load	Vascular calcification score (calcium score, lateral abdominal radiograph or echocardiography), review of cumulative calcium load	Low-calcium strategy, non-calcium binders, avoid high-dose vitamin D; investigational SNF472 or vitamin K	Cardiovascular events, mortality
Mineral-endocrine abnormalities	Systemic inflammation, low albumin, sarcopenia, gut dysbiosis	C-reactive protein, albumin, muscle mass, nutritional and toxin markers	Nutrition, dialysis adequacy, inflammation control; multi-target approach	Fracture, cardiovascular events, mortality, quality of life
clinical modifiers and treatment context	Persistent SHPT, glucocorticoid exposure, hypophosphatemia, rapid early bone loss	PTH, phosphate, DXA, glucocorticoid dose	Osteoporosis prevention, manage persistent SHPT, correct hypophosphatemia	Fracture

## 3 Epidemiology and clinical outcomes: from biochemical abnormality to fracture and cardiovascular death

The epidemiology of CKD-MBD is hard to pin down, because studies differ in their definitions, assays and CKD stages. The broad pattern is clear enough: as the estimated glomerular filtration rate (eGFR) falls, abnormalities of serum phosphate, PTH, FGF23 and vitamin D become steadily more common (4, 35). FGF23 tends to rise before overt hyperphosphatemia or a high PTH, an early compensatory move to hold phosphate balance (35). CKD-MBD is thus not a terminal event of advanced disease but an endocrine adaptation that begins early and slowly loses control.

Fracture is among the most important patient outcomes. Hip, vertebral and non-vertebral fractures are all markedly more frequent in advanced CKD and on dialysis, and the hospitalization, disability and death that follow a fracture exceed those in the general population (36–38). The excess risk reflects more than low bone mineral density; increased cortical porosity, disrupted microarchitecture, sarcopenia, falls, inflammation, malnutrition, diabetes, acidosis and drug exposure all contribute. DXA alone may understate fragility, because the cortical damage and disordered turnover typical of CKD are not fully captured by lumbar spine or hip bone mineral density (36, 37, 39–41).

Cardiovascular calcification is the second key outcome. Calcification of the coronary arteries, aorta, peripheral arteries and cardiac valves is common in CKD and tracks with cardiovascular events and mortality (20–25). It is an active process rather than passive salt deposition: vascular smooth muscle cells, exposed to high phosphate, high calcium, uremic toxins, oxidative stress and inflammation, undergo osteogenic transdifferentiation (21, 22), with Runx2, BMP, Wnt, Msx2, alkaline phosphatase, matrix vesicles and CPPs all implicated.

Across cohorts, serum phosphate, PTH and calcium relate to mortality in a U-shaped or otherwise non-linear way, so that lower is not reliably better (5–7). This is why KDIGO advises guiding treatment by continuous trends and integrated judgment rather than single thresholds (10, 42). Overusing calcium-containing binders or active vitamin D can lower PTH while adding calcium load and calcification risk; over-suppressing PTH can produce low-turnover bone disease; and leaving secondary hyperparathyroidism (SHPT) uncontrolled invites high-turnover disease, bone pain, fracture and resistance to anemia treatment. Good management does not chase a single normal value but holds a balance among mineral, bone-turnover and vascular-calcification risk in the context of an individual patient, which is precisely what the phenotype framework of Section 2 is meant to operationalize.

Four features together justify treating CKD-MBD as a problem in its own right: it starts early and lasts long; it strikes the skeleton and the cardiovascular system at once; its evidence rests mainly on surrogate endpoints, with little for hard ones; and its population is heterogeneous, yet advanced CKD patients are routinely kept out of general osteoporosis trials. These are the questions that frame the rest of this review.

## 4 Mechanisms linking CKD–MBD to bone-vascular risk: from biological plausibility to clinical outcomes

The mechanisms of CKD-MBD are intricate but follow one logic. When the kidney loses capacity to excrete phosphate and to activate vitamin D, the body compensates through FGF23, Klotho, PTH and bone remodeling; as CKD advances, that compensation loses balance and becomes entangled with inflammation, uremic toxins, a breached gut barrier, transdifferentiation of vascular smooth muscle cells and destruction of bone microarchitecture, each amplifying the others (18–21, 34, 43). These processes rarely act alone, and they map unevenly onto multidimensional bone-vascular framework of Section 2. Because a plausible mechanism is not the same as a proven benefit, this section also flags where each pathway sits on the evidence ladder; **Table 1** collects that grading for the mechanisms and interventions discussed here.

### 4.1 Phosphate retention: a major pathophysiological driver of CKD-MBD

Phosphate is the factor that links kidney, bone and vessel, and it dominates the high-phosphate/high-PTH phenotype. In health, intestinal absorption, skeletal storage and renal excretion hold it in balance. In early CKD, even a normal serum phosphate can be deceptive: nephron loss raises the excretory load on each surviving nephron, and FGF23 and PTH climb to suppress the proximal tubular cotransporters NaPi-IIa and NaPi-IIc and drive phosphate into the urine (35, 44, 45). A normal value may therefore mark not true homeostasis but a fragile balance bought with high FGF23 and high PTH.

Sustained phosphate excess then feeds the syndrome along several routes. It stimulates osteocyte secretion of FGF23, which suppresses 1α-hydroxylase and lowers 1,25(OH)_2_D (44, 45). It drives phosphate into vascular smooth muscle cells through transporters such as PiT-1, switching on Runx2 and the osteogenic program that produces vascular calcification (21, 22). Bound to calcium, it forms primary and secondary CPPs that injure endothelium and promote calcification in an inflammatory setting (26–28). And, by raising PTH and FGF23, it acts indirectly on bone turnover and cardiac structure as well.

In the clinic, phosphate management is often reduced to restricting high-phosphate foods and prescribing binders, but the source of the phosphate matters more than the total. Organic phosphate in natural foods is absorbed relatively poorly, and phytate lowers absorption from plant foods further, whereas the inorganic phosphate added to processed foods is absorbed almost completely and is hard for patients to spot on a label. Advice should therefore aim less at total intake than at cutting ultra-processed foods with phosphate additives, while protecting high-quality protein so as not to provoke protein-energy wasting.

The binder classes differ in ways that matter for long-term care, in calcium load, gastrointestinal tolerability, iron balance, pill burden and the strength of outcome data (46–53). Calcium-containing binders are cheap and effective but add calcium load and calcification risk with prolonged use; non-calcium binders such as sevelamer and lanthanum carbonate spare calcium but carry gastrointestinal side effects and cost that erode adherence; iron-based binders lower phosphate while repleting iron, at the price of monitoring iron stores and tolerance (46–53). Inhibitors of intestinal phosphate absorption such as tenapanor add another option (52). It is worth stating plainly, however, that phosphate binders lower serum phosphate, but adequately powered randomized evidence for mortality or cardiovascular benefit remains lacking or inconsistent (49).

### 4.2 The FGF23–Klotho axis: from adaptive regulation to a marker of CKD-MBD risk

FGF23, secreted mainly by osteocytes and osteoblasts, is a central endocrine regulator of phosphate and vitamin D metabolism (44, 54). In early CKD it raises urinary phosphate excretion by lowering proximal tubular NaPi-IIa/IIc, and it reduces 1,25(OH)_2_D by inhibiting 1α-hydroxylase and inducing 24-hydroxylase (35, 44). In the short term this protects a normal serum phosphate. Sustained over years, however, a high FGF23 is associated with, and may contribute to, vitamin D deficiency, PTH imbalance, myocardial hypertrophy and death, although these are complex outcomes with many determinants and the causal weight of FGF23 itself remains uncertain (55–57).

Klotho is the co-receptor that makes the canonical actions of FGF23 possible, and its loss is one of several drivers of the rise in the hormone. Renal Klotho expression falls early in CKD and blunts FGF23 signaling, but the elevation of FGF23 in CKD is multifactorial, reflecting phosphate load, declining calcitriol, PTH, inflammation and iron status as well as Klotho deficiency, and should not be reduced to a single linear chain (54, 58–61). Because Klotho also has antioxidant, phosphate-lowering and endothelial-protective actions, its loss reaches beyond phosphate handling into vascular calcification and cardiovascular remodeling (58, 59, 62). As a clinical biomarker, though, soluble Klotho remains limited by non-standardized assays, sensitivity to sample handling and inconsistent findings (62).

Whether FGF23 should be targeted directly remains uncertain. Although reducing excessive FGF23 may theoretically mitigate myocardial hypertrophy and mineral dysregulation, FGF23 also serves as a compensatory mechanism for enhancing phosphate excretion in CKD. Broad inhibition could therefore aggravate phosphate retention, hyperphosphatemia, and vascular calcification. At present, a more cautious strategy is to attenuate FGF23 elevation indirectly by reducing phosphate burden, correcting vitamin D deficiency where appropriate, controlling inflammation, and preserving Klotho signaling. Direct FGF23 suppression should be considered only after the underlying phosphate balance and the potential loss of this adaptive response have been carefully assessed. The axis is thus not a matter of simple up or down. Early elevation is compensatory; later, against Klotho deficiency, a persistent phosphate load and ongoing inflammation, the same elevation shades into an adverse signal. Its meaning depends on CKD stage, serum phosphate, PTH, vitamin D therapy and cardiovascular background. In terms of evidence, the axis is strong on mechanism and observational association but has, so far, no intervention that improves hard endpoints (**Table 1**).

### 4.3 The vitamin D–PTH axis and secondary hyperparathyroidism

Vitamin D and PTH are the two most clinically actionable levers in CKD-MBD, and their derangement defines the high-turnover phenotype. As CKD advances, renal 1α-hydroxylation declines, rising FGF23 further suppresses 1,25(OH)_2_D, intestinal calcium absorption falls, and the resulting hypocalcemia drives PTH secretion. High phosphate, low vitamin D, low calcium and FGF23 resistance together push parathyroid cell proliferation, producing SHPT (63–65).

A high PTH is not always pathological. A modest early rise helps excrete phosphate and defend calcium; but a chronic, excessive or unchecked rise brings high-turnover bone disease, bone pain, cortical porosity, fracture, and disturbances of anemia and nutrition (31, 66, 67). Over-suppression carries the opposite danger, since low-turnover or adynamic bone disease weakens the skeleton’s buffering of a calcium load and may accelerate vascular calcification. PTH should therefore be read against its own trend and against calcium, phosphate, bone-specific alkaline phosphatase, imaging and symptoms, never from a single value (10, 42).

The therapeutic tools each cut both ways, and here the gap between lowering a marker and improving an outcome is especially instructive. Active vitamin D and its analogues suppress PTH synthesis and parathyroid hyperplasia but can raise intestinal calcium and phosphate absorption enough to cause hypercalcemia or hyperphosphatemia; KDIGO accordingly advises against their routine use in non-dialysis CKD and reserves them for CKD G4-G5 patients with severe and progressive SHPT once modifiable factors have been corrected (10, 68). Calcimimetics lower PTH through the calcium-sensing receptor, and two randomized trials showed that etelcalcetide lowers PTH effectively in dialysis patients, with intravenous dosing that eases adherence (64, 69); yet the same literature is candid that a fall in PTH is not proof of clinical benefit. The EVOLVE trial of cinacalcet did not significantly reduce death or major cardiovascular events in its unadjusted intention-to-treat analysis, a result that should temper any assumption that suppressing PTH necessarily improves outcomes (69). Calcimimetics can also provoke hypocalcemia and gastrointestinal upset and need monitoring where vitamin D, calcium intake or bone turnover is already low.

When medical therapy fails in severe SHPT, parathyroidectomy remains a key option. Meta-analysis in dialysis patients shows marked improvement in PTH and several biochemical markers after surgery, and a possible survival benefit, although this evidence is observational and operative approach, perioperative hypocalcemia, hungry bone syndrome and late recurrence all demand individualized judgment (67). It helps to see SHPT treatment as a continuum: correct vitamin D deficiency and phosphate load early, use active vitamin D, calcimimetics and non-calcium binders judiciously in mid-course, and evaluate surgery once hyperplasia becomes nodular and drug-resistant.

### 4.4 Skeletal manifestations of CKD-MBD: bone turnover, renal osteodystrophy, and skeletal fragility

CKD bone disease is not the same as ordinary osteoporosis. Osteoporosis is defined by reduced bone mass and disrupted microarchitecture, whereas ROD additionally encompasses abnormalities in bone turnover and mineralization (8, 31). Within the bone turnover/mineralization domain, high-turnover states occur in SHPT, in which both osteoclastic and osteoblastic activities are elevated despite deterioration of bone structural quality. By contrast, low-turnover states may be driven by multiple clinical modifiers including diabetes mellitus, advanced age, excessive PTH suppression, high calcium load, and long-term dialysis; in this setting, bone remodeling becomes impaired, microdamage accumulates without repair, and adynamic bone disease represents the extreme manifestation of low-turnover bone status. Mixed ROD features concurrent derangements of both mineralization and bone turnover. Notably, CKD-associated osteoporosis may develop across the full spectrum of these bone turnover states, and therapeutic decision-making should be guided by these distinct bone statuses rather than discrete composite phenotypes.

That separation drives treatment. In high-turnover disease, controlling SHPT and phosphate load can relieve bone pain and cortical damage. In low-turnover disease, pressing on with calcium, active vitamin D or calcimimetics may suppress remodeling further and add to fragility and calcification risk, and antiresorptives such as bisphosphonates and denosumab risk over-suppression on the same background. In advanced CKD, then, the turnover state should be established as far as possible before osteoporosis is treated (32, 39–41, 70, 71).

Because bone biopsy, the gold standard for ROD, cannot be done routinely in most centers, turnover has in practice to be inferred from a multimodal, individualized assessment. The tools available for that inference, and a corresponding management pathway, are examined in Section 5 (**Table 3**, **Figure 1**).

**Table 3:** Assessment tools in CKD-MBD and the clinical decisions they inform.

Tool	Decision it informs	Advantages	Limitations
Serum calcium, phosphate, PTH	Mineral-load and SHPT trajectory; treatment titration	Highly accessible; suited to serial monitoring	Affected by diet, drugs, dialysis and timing; cannot judge bone turnover alone
Bone-specific alkaline phosphatase, P1NP, TRAP5b	High- versus low-turnover tendency	Complement PTH in judging turnover	Thresholds not fully unified; need clinical context
DXA bone mineral density	Fracture-risk prediction	Predicts fracture; widely available	Cannot distinguish ROD subtypes, turnover or mineralization
HR-pQCT	Cortical and trabecular structural damage (research; not for routine decisions)	Resolves microarchitecture	Low availability; standardization and longitudinal thresholds unvalidated
Vascular calcification scoring/imaging	Cardiovascular risk and calcium-load strategy	Identifies the calcification-principal management priority	Screening frequency and treatment-response reading not standardized
T50/CPPs	Calcification propensity (research; not for routine decisions)	Functional, integrative readout	Standardization and decision thresholds unsettled
Bone biopsy	Definitive turnover, mineralization and mass	Gold standard for ROD	Invasive, technically demanding, hard to use routinely

**Figure 1:**
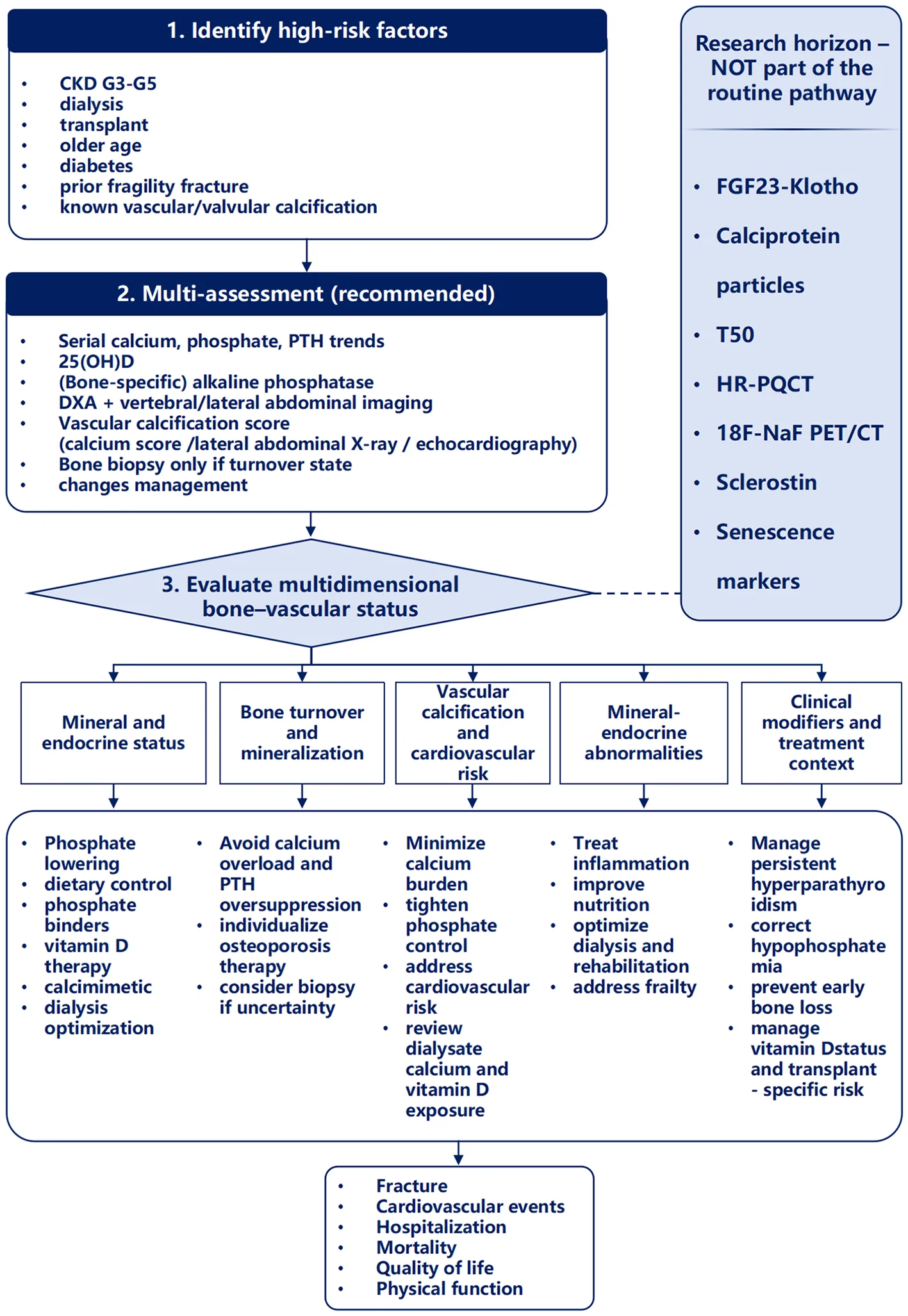
Risk stratification and phenotype-directed management of CKD-MBD. A pragmatic five-step approach to CKD-MBD assessment. High-risk clinical contexts are identified first, followed by routine biochemical assessment, DXA with vertebral-fracture assessment, separate evaluation of abdominal aortic and valvular calcification, and selective use of bone biopsy when the turnover state would alter management. Patients are assessed across overlapping domains rather than assigned to mutually exclusive phenotypes. Five heuristic working patterns—high-phosphate/high-PTH, low-turnover/diabetic, calcification-dominant, inflammation–malnutrition, and post-transplant—may help organize clinical priorities but are not validated diagnostic classes. Treatment is individualized, and follow-up is based on fractures, cardiovascular events, hospitalization, mortality, quality of life, and physical function rather than biochemical normalization alone. The shaded panel lists emerging, not-yet-validated research markers; their clinical readiness is summarized in **Table 1**.

Non-invasive work on ROD is moving from replacing biopsy toward reducing uncertainty. HR-pQCT quantifies cortical porosity, trabecular thickness and microarchitecture and suits the structural questions of CKD-associated osteoporosis, and time-lapse HR-pQCT goes further, estimating local formation and resorption from serial change. 18F-NaF PET/CT reads metabolic activity from bone uptake kinetics and has been compared with histomorphometry to predict turnover on dialysis. Neither replaces biopsy yet, but where routine biopsy is impossible, a model that combines PTH, bone-specific alkaline phosphatase, DXA, HR-pQCT and 18F-NaF PET/CT offers a workable way to make treatment safer (72–77).

The real obstacle in ROD is less the pathological classification than the effect of diagnostic uncertainty on decisions. Biochemistry stands in for pathology but cannot separate high from low turnover; DXA predicts fracture but cannot define turnover; biopsy is accurate but not scalable. Resolving that tension is one of the field’s central tasks.

### 4.5 Wnt/β-catenin, sclerostin and restricted bone formation

The Wnt/β-catenin pathway is a principal regulator of osteoblast differentiation and bone formation. Sclerostin, an osteocyte product, binds LRP5/6 and blocks canonical Wnt signaling to restrain osteogenesis, and DKK1 restrains it by a similar route (78–80). In CKD-MBD, both are linked to low turnover, restricted formation and vascular disease, though the literature is not uniform. Sclerostin is reported to be elevated in dialysis patients and positively correlated with bone mineral density, which likely reflects osteocyte mass, reduced clearance or a low-turnover state rather than a straightforward “more sclerostin, more bone” (81).

The pathway is also where bone and vessel meet. The same pathway operates in the vessel wall during uremic calcification (21, 22, 78–80). In principle, a sclerostin inhibitor such as romosozumab both builds bone and curbs resorption, which could help patients with poor bone formation; but because sclerostin’s role in the vessel wall is not simple, its use in advanced CKD calls for careful appraisal of cardiovascular risk (13, 82, 83).

One point deserves care, since it is easy to invert. Sclerostin’s block on Wnt lowers bone formation rather than raising bone mass, and anti-sclerostin therapy works by lifting that block; the concern in CKD G4-G5D and on dialysis is not the mechanism but the thinness of the safety evidence.

### 4.6 Vascular calcification, CPPs and osteogenic transdifferentiation of vascular smooth muscle cells

Vascular calcification in CKD comes early, moves fast and carries a poor prognosis, and it defines the calcification-principal management priority. Unlike ordinary atherosclerosis, it often involves both intima and media and reflects high phosphate, high calcium, inflammation, oxidative stress, uremic toxins, vitamin K deficiency and a shortfall of calcification inhibitors (20–30). Under these pressures, vascular smooth muscle cells shed their contractile phenotype for an osteogenic or chondrogenic one, expressing Runx2, alkaline phosphatase and osteocalcin while shedding matrix vesicles that seed calcium–phosphate deposition.

CPPs have reframed how the process is understood. Calcium and phosphate bind inhibitors such as fetuin-A to form primary CPPs that keep free ions in solution; in a high-phosphate, inflammatory, uremic milieu these mature into secondary CPPs that are more crystalline and more strongly pro-inflammatory and pro-calcific (26–28). The T50 test reads serum calcification propensity and may become a functional predictor of calcification and mortality,once its standardization and decision thresholds are settled (27). In dialysis cohorts, fetuin-A has also been linked to vascular calcification and fracture risk, which makes it useful as a mechanistic marker rather than a stand-alone treatment target (84).

Current management generally focuses on addressing modifiable contributors to calcification, including limiting excessive calcium and phosphate exposure, avoiding sustained hypercalcemia and hyperphosphatemia, ensuring adequate dialysis delivery, and treating underlying inflammation and malnutrition when present. While documented vitamin K or magnesium deficiency may warrant repletion, supplementation of vitamin K or magnesium cannot be regarded as established therapies to treat or prevent vascular calcification; they remain emerging, investigational strategies for this indication (29, 30, 85–87). In a randomized phase 2b trial among hemodialysis patients, SNF472, an inhibitor of hydroxyapatite crystallization, was linked to slower progression of coronary and aortic-valve calcification. Nevertheless, its beneficial effects on hard clinical endpoints including cardiovascular events and all-cause survival have not been formally established, and SNF472 is therefore still investigational. Collectively, these observations have expanded the research agenda beyond conventional phosphate-lowering strategies to encompass direct inhibition of hydroxyapatite crystal formation and modulation of circulating calcification propensity. Even so, the clinical utility and real-world relevance of these novel therapeutic targets for routine patient care remain to be fully determined.

### 4.7 Inflammation, gut microbiota, uremic toxins and cellular senescence: the systemic background that amplifies the bone–vascular axis

CKD-MBD is increasingly read not as a purely endocrine disease but as an immunometabolic disorder of a gut–kidney–bone axis, and this background defines the inflammation–malnutrition phenotype. Uremia disturbs the gut microbiota, breaches the intestinal barrier and allows endotoxin translocation, sustaining systemic inflammation (18, 19, 88, 89). Cytokines such as IL-6, TNF-α and IL-1β act on osteocytes, osteoblasts, osteoclasts and vascular smooth muscle cells to promote resorption, suppress formation and speed calcification (18, 19, 90–93).

Gut dysbiosis ties these strands together. A higher intestinal urea load, low dietary fiber, phosphate binders and iron agents, antibiotics and a damaged barrier together shift the microbiota toward protein fermentation, raising indoxyl sulfate, p-cresyl sulfate, trimethylamine N-oxide (TMAO) and endotoxin (18, 88, 89).

Retained toxins and cellular stress form the background against which all of this plays out. As function declines, indoxyl sulfate, p-cresyl sulfate, guanidino compounds and advanced glycation end products accumulate and reach bone and vessel cells through oxidative stress, mitochondrial dysfunction, endothelial injury and inflammation (89, 90, 93). Indoxyl sulfate suppresses osteoblastic differentiation, promotes oxidative stress and tracks with calcification and cardiovascular risk (89). Advanced glycation end products cross-link bone collagen abnormally and blunt its toughness, so bone strength can fall even when density does not, a point that weighs heavily in diabetic CKD.

Oxidative stress works as an amplifier. High phosphate, uremic toxins, cytokines and dialysis-related stimuli all raise reactive oxygen species. *In vitro* and in animals, these species push vascular smooth muscle cells toward the osteogenic phenotype and strengthen Runx2 and BMP signaling while impairing endothelial function and nitric oxide (21, 22, 88, 89); in bone, they suppress osteoblast differentiation and promote osteocyte apoptosis, indirectly favoring resorption (90, 93).

Cellular senescence is a newer thread, and the human evidence is still thin. Klotho deficiency itself carries an aging-like phenotype (58, 59, 62), and vascular smooth muscle cells, endothelium and immune cells in CKD may take on a senescence-associated secretory phenotype (SASP), that may amplify calcification and bone loss; the human evidence, however, remains preliminary and carries no current clinical action.

The caution that applies to this whole module is one of evidence level. Interventions aimed at the microbiota, inflammation and senescence are still at the stage of mechanism and early trials, and probiotics, fiber and similar strategies have not yet been shown to move the hard endpoints of CKD-MBD. **Table 1** places these pathways at the low-readiness end of the ladder deliberately: they are promising targets and useful explanations for the heterogeneity of the syndrome, not established treatments. In a word, CKD-MBD drives maladaptive bone–vascular crosstalk through mineral–endocrine imbalance, inflammation, and cellular dysfunction, increasing fracture, calcification, cardiovascular events, and mortality (**Figure 2**).

**Figure 2:**
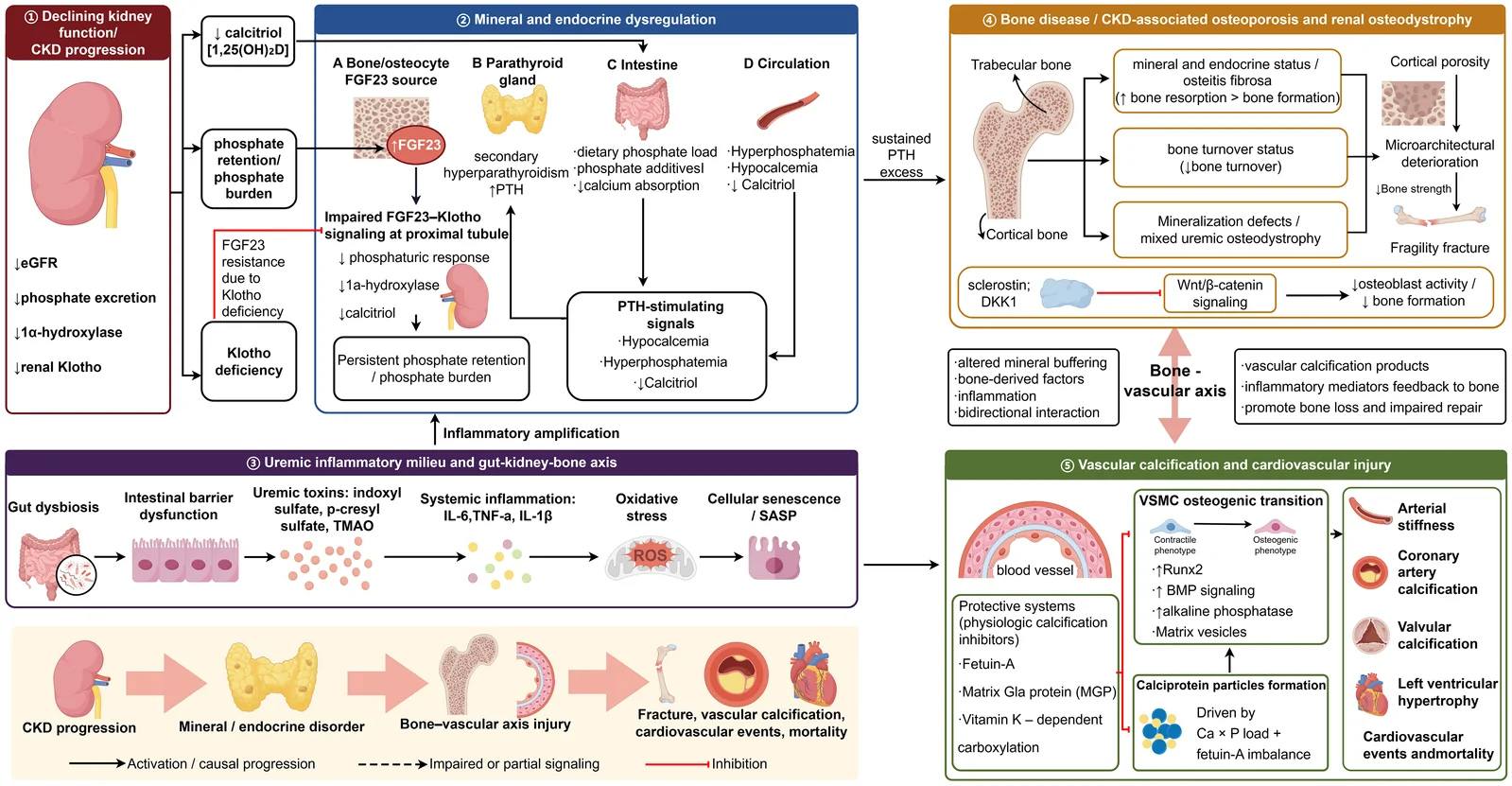
Integrated pathophysiology of CKD-MBD and the bone–vascular axis. Declining kidney function initiates phosphate retention, impaired calcitriol synthesis and Klotho deficiency; compensatory FGF23 and PTH responses preserve mineral balance early but may become maladaptive as CKD progresses. These mineral-endocrine changes interact with gut dysbiosis, uremic toxins, inflammation, oxidative stress and cellular senescence to amplify renal osteodystrophy, CKD-associated osteoporosis and vascular smooth muscle cell osteogenic transition, and the resulting bone-vascular crosstalk links microarchitectural bone damage, fracture, vascular and valvular calcification, cardiovascular events and mortality. Solid arrows, activation or causal progression; dashed arrows, impaired or partial signaling; red inhibitory lines, suppression.

## 5 Diagnosis and risk stratification: an evidence-informed framework for clinical decisions

To keep everyday care usable, we separate a small clinical core from the research horizon and base management on the core alone. (1) Track serial calcium, phosphate and PTH rather than single values, adding 25(OH)D and (bone-specific) alkaline phosphatase to help separate high- from low-turnover disease. (2) Assess fracture risk with DXA and vertebral or lateral abdominal imaging, reserving bone biopsy for the uncommon case in which the turnover state changes management. (3) Screen for vascular or valvular calcification with a calcium score, lateral abdominal radiograph or echocardiography. (4) Assign the patient to a dominant bone–vascular phenotype and treat accordingly, judging success by fracture, cardiovascular events, hospitalization, mortality, quality of life and physical function rather than by the numbers a treatment normalizes. By contrast, the FGF23–Klotho axis, CPPs, T50, HR-pQCT, 18F-NaF PET/CT, sclerostin and senescence markers are mechanistically informative but are not part of this routine pathway and should not drive individual treatment decisions until they are prospectively validated (Section 8; **Table 1**).

### 5.1 Dynamic monitoring: why trend matters more than a single value

CKD-MBD markers are restless. Serum phosphate moves with recent diet, the interdialytic interval, adherence and the timing of the draw; calcium with albumin, acid–base status, calcium agents, active vitamin D and dialysate calcium; PTH with biological variation and the choice of assay; and bone turnover markers with renal clearance, inflammation and fracture healing. Sound management therefore weighs the trend more heavily than any single abnormal value, and KDIGO advises reading calcium, phosphate and PTH together and setting monitoring frequency to CKD stage and the degree of abnormality (10, 42).

What the trend reveals is direction, and direction often points to a phenotype. A steady rise in phosphate with rising FGF23 and PTH marks a growing phosphate load and progressing SHPT, the high-turnover phenotype in evolution. A rapid fall in PTH with a falling bone alkaline phosphatase warns of over-suppression toward low turnover. Repeatedly high calcium alongside established calcification points to the calcification-principal management priority and should prompt a fresh look at calcium-containing binders, active vitamin D and dialysate calcium. A persistently very high PTH with bone pain and a high alkaline phosphatase raises severe SHPT and high-turnover disease.

Ordinary reference ranges also travel poorly into CKD. A moderate rise in PTH may be compensatory, and suppressing it into the general-population range is not necessarily helpful; a high-normal phosphate can hide a markedly elevated FGF23 and a rising load; and a normal calcium does not rule out an excess that is depositing in vessel or soft tissue. For these reasons, CKD-MBD is closer to risk medicine than to the correction of abnormal values.

Electronic records and machine learning methods may help formalize this. A system that integrates long-term phosphate, calcium and PTH with drug doses, dialysis prescription, fracture history, calcification imaging and nutrition could flag high-risk trajectories and prompt adjustment, which would suit dialysis units and chronic-disease programs, provided such models are shown prospectively to improve outcomes rather than merely to predict them.

### 5.2 From measurement to decision: assessment priorities across settings and phenotypes

Clinical priorities should be individualized according to CKD stage, treatment setting, serial biochemical trends, skeletal risk, and evidence of vascular or valvular calcification. Although different settings may be associated with different patterns of CKD-MBD, no single biochemical abnormality is sufficient to define a specific phenotype. In non-dialysis CKD G3–G4, assessment should focus on identifying and addressing potentially modifiable abnormalities early, including documented vitamin D deficiency, a progressive rise in PTH, and increasing or high-normal phosphate when interpreted in the context of serial measurements and the overall CKD-MBD profile. Nutritional vitamin D repletion may be considered when deficiency is present, whereas unnecessary calcium loading should be avoided. Fracture risk should be assessed in parallel using established risk factors, including diabetes, postmenopausal status, glucocorticoid exposure, and prior fracture.

In CKD G5D, management is more complex because phosphate control, SHPT, calcium exposure, vascular or valvular calcification, and bone turnover must be considered together with the dialysis prescription, dietary intake, adherence, pill burden, and substantial biological variability. Persistently elevated PTH together with elevated alkaline phosphatase may support a high-turnover state, particularly when accompanied by compatible skeletal symptoms or fractures, but these findings are not diagnostic in isolation. Conversely, persistently low PTH, especially in the presence of diabetes, older age, substantial calcium exposure, or established calcification, may raise concern for low-turnover bone disease or a calcification-prone clinical pattern. Such interpretations should be based on serial measurements and the broader clinical context, with additional evaluation considered when the diagnosis would alter management.

When anti-osteoporotic drugs are on the table, the useful question is not the T-score but three others: is the fracture risk genuinely high, does the turnover state allow an antiresorptive or a bone-forming agent, and will treatment add a risk of hypocalcemia, hypercalcemia or cardiovascular harm. Patients in CKD G1-G3 without evident CKD-MBD can be managed by general osteoporosis guidelines; those in CKD G4-G5D need more specialist input.

Where vascular or valvular calcification is already present, the priority is to lower calcium and phosphate load and to avoid combinations that speed calcification, which means particular caution with calcium-containing binders, higher-calcium dialysate and high-dose active vitamin D. A coexisting low PTH and low turnover only sharpen the concern, since bone then buffers minerals poorly and the vessel is left more exposed.

### 5.3 Multimodal assessment tools: advantages and limitations

The diagnostic difficulty is a mismatch of what is easy to measure against what patients face. Calcium, phosphate, PTH and vitamin D are readily available, yet the real stakes are fracture and cardiovascular events, and no single marker captures that risk, which is why KDIGO recommends integrated assessment across CKD stage, marker trends, bone imaging, calcification and clinical presentation (10, 33, 42). The value of any given tool is best judged by the decision it informs, and **Table 3** is organized on that principle.

Biochemistry remains the foundation. Calcium and phosphate index mineral load but move with albumin, diet, dialysis, drugs and timing; PTH indexes parathyroid activity and turnover but predicts weakly in its mid-range; bone-specific alkaline phosphatase reflects osteoblastic activity somewhat better and helps sort high from low turnover; 25(OH)D gauges nutritional vitamin D; and FGF23 and Klotho, are mechanistically informative but remain research tools and should not drive routine clinical decisions (10, 42, 70–72, 77, 94).

Imaging is increasingly important for assessing skeletal fragility in CKD-associated osteoporosis, but skeletal fragility should be distinguished from bone-turnover status. DXA provides an accessible measure of areal bone mineral density and has prognostic value for fracture risk in CKD G3-G5D (10, 36–38, 40, 41). However, it cannot distinguish cortical from trabecular bone, characterize microarchitecture, or directly assess bone turnover; therefore, low BMD should not be equated with low-turnover bone disease. HR-pQCT provides more detailed information on cortical porosity, trabecular number, and microarchitecture, but its cost, limited availability, and limited clinical validation restrict routine use (39). Vertebral imaging can identify clinically silent compression fractures, while FRAX may provide additional fracture-risk stratification, although its calibration and performance are less certain in advanced CKD and it does not fully account for CKD-MBD-specific abnormalities (95).

Vascular calcification can be scored by coronary artery calcium, lateral abdominal radiography, ultrasound, CT or valvular assessment (24, 25). KDIGO treats known vascular or valvular calcification as a marker of high cardiovascular risk and a reason for extra caution with calcium-containing binders and vitamin D (10, 42). In practice, screening is not standardized, and when to image, how often to repeat it, and how to let progression change treatment remain open.

Bone biopsy is still the gold standard, and it earns its place in complex cases where the decision turns on the turnover state (32, 70, 96, 97). Since few centers can offer it routinely, the practical path is a multimodal model that weighs CKD stage, PTH trend, bone-specific alkaline phosphatase, DXA or vertebral imaging, calcification, fracture history, diabetes and drug exposure together. Adding bone turnover markers, calcification propensity, machine-learning image analysis and dynamic record-based risk models to that mix could sharpen individualized care further.

## 6 Therapeutic target-directed, evidence-informed management of CKD-MBD

After risk stratification, treatment should be directed toward the dominant CKD-MBD phenotype and evaluated against both biochemical and patient-important outcomes. In general, evidence is stronger for lowering serum phosphate and PTH than for reducing fractures, cardiovascular events, vascular calcification, or mortality. Management should therefore aim to control mineral disturbances while avoiding excessive calcium exposure, treatment-related complications, and nutritional harm. The evidence grades and major limitations of each intervention are summarized in **Tables 1** and **4**.

**Table 4:** Phenotype-informed management of CKD–MBD: evidence, uncertainty, and clinical boundaries.

Phenotype	Treatment strategies	Attentions
bone turnover status (high-PTH)	Dietary education, reducing phosphate additives, phosphate binders, optimizing dialysis	Avoid malnutrition; calcium-containing binders add calcium load and calcification risk; no RCT shows a mortality benefit
bone turnover status (Low-turnover/diabetic)	Active vitamin D or analogues (selective use), calcimimetics, parathyroidectomy	Reserve active vitamin D for severe progressive SHPT; PTH lowering is not proven to improve hard outcomes; watch hypocalcemia and hungry bone syndrome
skeletal fragility and structural consequences	Bisphosphonates, denosumab, teriparatide, romosozumab, sequential therapy	Evidence thin in advanced CKD; assess turnover first; denosumab needs a strict hypocalcemia-monitoring routine
Mineral-endocrine abnormalities	Lower calcium and phosphate load, non-calcium binders, vitamin K/magnesium, SNF472	Most interventions lack hard-endpoint evidence and should not be over-interpreted
Post-transplant bone disease	Nutrition, dialysis adequacy, inflammation control; osteoporosis prevention and SHPT/phosphate management after transplantation	Multi-target strategies are unproven on hard endpoints; individualize

### 6.1 Management of phosphate burden and secondary hyperparathyroidism, with considerations for probable or confirmed high-turnover bone disease

This phenotype, dominated by phosphate retention and SHPT, is where mineral-load control and PTH-lowering therapy belong. Diet is the foundation of managing hyperphosphatemia, but nutrition sets its boundary: over-restricting protein invites protein–energy wasting, so advice should turn on the source of phosphate, fewer processed foods carrying inorganic additives, a preference for a lower phosphate-to-protein ratio, and better food-label literacy, all of which the evidence supports building into individualized education (98–108). Whole grains and legumes carry more phosphate but bind much of it as poorly available phytate, so plant-leaning choices can lower the load while protecting protein quality (105, 106). Binder choice follows phosphate, calcium, PTH, calcification and tolerance: calcium-containing binders are poorly suited to long-term high-dose use where there is hypercalcemia, a low PTH, marked calcification or a high calcium load, whereas non-calcium and iron-based binders spare calcium and the iron-based agents may reduce the need for intravenous iron and erythropoiesis-stimulating agents, at the cost of watching iron parameters (46–53). Binder therapy improves biochemical control, but its effect on patient-important outcomes remains uncertain. More frequent or prolonged dialysis may enhance phosphate removal, but should be individualized and used together with dietary and binder-based strategies.

In patients with non-dialysis CKD G3–G5, potentially reversible drivers of SHPT, including hyperphosphatemia, vitamin D deficiency, and clinically relevant hypocalcemia, should be assessed and corrected where appropriate. Active vitamin D or its analogues should not be used routinely and may be reserved for severe, progressive SHPT in CKD G4–G5 after modifiable factors have been addressed (10, 68, 109). On dialysis, a markedly high PTH can be met with active vitamin D, a calcimimetic or both, the aim being not to force PTH into the normal range but to avoid either extreme elevation or over-suppression. Calcimimetics lower PTH without, as a rule, adding calcium or phosphate absorption, which suits a high PTH with a tendency to hypercalcemia (63, 64), and intravenous etelcalcetide lightens the oral pill burden (110). Nevertheless, the EVOLVE trial showed that cinacalcet lowered PTH without significantly reducing mortality or cardiovascular events, underscoring the uncertain translation of biochemical improvement into clinical benefit (69). Parathyroidectomy should be considered in patients with severe, persistent SHPT, suspected nodular hyperplasia, and inadequate response or intolerance to medical therapy, particularly when bone symptoms, persistent mineral abnormalities, or progressive calcification are present. Postoperative hypocalcemia and hungry bone syndrome should be anticipated and managed with planned monitoring and calcium/vitamin D replacement (67).

### 6.2 Management of skeletal fragility and CKD-associated osteoporosis, with diabetes and low-turnover bone disease as clinical considerations

The low-turnover phenotype inverts several of the priorities above. Here the danger is over-treatment: continued calcium, active vitamin D or calcimimetics can suppress an already sluggish skeleton further, and antiresorptives compound the problem, so the first step is often to reduce calcium and vitamin D load rather than add to it. CKD-associated osteoporosis nonetheless remains among the hardest problems in the field, because fear of ROD and of drug safety long kept effective agents from patients who needed them. European consensus and recent reviews take a firmer line: the fracture burden in CKD G4-G5D is heavy, and imperfect evidence is not a reason to abandon anti-fracture treatment, provided turnover, calcium–phosphate metabolism, PTH and calcification risk are assessed first (11–17, 111–113).

The agents differ in how far they carry into advanced CKD, and the evidence thins as kidney function falls (**Table 1**). Bisphosphonates, cleared mainly by the kidney, are limited there (17, 114); patients in CKD G1-G3 without evident CKD-MBD can largely follow general osteoporosis principles, while CKD G4-G5D calls for careful weighing of low-turnover and nephrotoxicity risk, with biopsy or specialist input where warranted. Denosumab is not renally cleared and so appeals in reduced function, but the risk of severe hypocalcemia rises steeply in advanced CKD and on dialysis, and the FDA has added a boxed warning that includes hospitalization and life-threatening events (14, 15, 115). Its use therefore demands a defined safety routine: correct vitamin D deficiency and hypocalcemia before dosing; recognize severe SHPT or high-turnover states because of the risk of profound post-dose hypocalcemia; use caution in suspected adynamic bone disease, where further suppression of remodeling may be harmful; monitor serum calcium closely in the first weeks after each dose; and treat dialysis patients with particular caution. Bone-forming agents such as teriparatide may suit low-turnover disease or a genuinely high fracture risk on a background of low or normal PTH, but not uncontrolled severe SHPT (116, 117). Romosozumab, which inhibits sclerostin while both building bone and curbing resorption, raised bone density and lowered vertebral fracture risk in women with osteoporosis and mild-to-moderate CKD (13), but the evidence thins out in advanced CKD, on dialysis and at high cardiovascular risk, and sequential regimens cannot be copied wholesale from general osteoporosis because coming off denosumab can bring rebound turnover, hypocalcemia or bone loss (15, 82, 83, 115–121). The diabetic variant of this phenotype deserves special mention: advanced glycation end products degrade bone quality so that fracture risk can be high even when density is not, which makes turnover markers and imaging, rather than density alone, the better guide.

Non-drug measures count for as much across this phenotype. CKD patients often carry sarcopenia, falls, malnutrition, metabolic acidosis and polypharmacy, and exercise and balance training, correction of vision and home hazards, better protein and vitamin D status, treatment of acidosis, and fewer sedatives and excessive antihypertensives can all lower fracture risk. After a vertebral or hip fracture, a fracture-liaison service joining nephrology, orthopedics, endocrinology, rehabilitation and nutrition should be in place.

### 6.3 Management of vascular calcification risk as a coexisting domain

Where vascular or valvular calcification is established, the calcium load itself becomes the thing to manage, and no drug has yet been shown convincingly to reverse CKD calcification and improve hard endpoints. Strategy rests on controlling phosphate load, avoiding hypercalcemia, limiting overuse of calcium-containing binders, keeping PTH in range, correcting vitamin D deficiency, improving dialysis, and controlling inflammation and malnutrition (20–30, 85–87). Non-calcium binders and avoidance of unnecessary calcium loading, including individualized review of dialysate calcium, are preferable here, and high-dose active vitamin D is best avoided.

The emerging options remain investigational and should be presented as such. Vitamin K carboxylates matrix Gla protein, and a shortfall of carboxylated MGP may weaken the vessel’s defense against calcification (85, 86),while lower serum magnesium has been associated with denser coronary calcification in CKD (87);but the randomized vitamin K trials disagree, some improving dp-ucMGP or slowing progression and others failing to move imaging calcification, so future trials should stratify by baseline vitamin K status, dp-ucMGP, calcification site, dialysis status, calcium load and follow-up length rather than simply test whether vitamin K is given (122–129). SNF472 takes the more direct route of inhibiting hydroxyapatite crystallization and slowed coronary and aortic-valve calcification in a phase 2b trial on hemodialysis (29); should it prove to cut events or extend survival it would reshape the field, but until then it is a promising investigational strategy, not a standard of care. Calcification propensity testing and CPPs study, meanwhile, offer a risk readout that plain calcium and phosphate cannot, linking mineral load, inflammation and cellular transdifferentiation, and they may eventually tell which calcification is still modifiable and which is only a scar of past injury (26–28, 124).

### 6.4 Inflammation–malnutrition as a systemic modifier and post-transplant bone disease as a special clinical context

The inflammation–malnutrition phenotype has no single drug target and is managed by addressing its drivers: nutritional support that avoids protein–energy wasting, adequate dialysis, control of inflammation, and attention to the gut–toxin axis. The multi-target interventions that follow from the mechanisms of Section 4.7, high-fiber diets, prebiotics, intestinal adsorbents and anti-inflammatory strategies, are biologically reasonable but unproven on hard endpoints, and should be offered as such rather than as established therapy. The post-transplant phenotype, by contrast, shifts the emphasis again: long-standing pre-transplant CKD-MBD, persistent parathyroid hyperplasia, glucocorticoids, hypophosphatemia and tubular phosphate loss combine to threaten bone, some recipients lose bone rapidly in the early months, and care moves toward preventing osteoporosis, managing persistent SHPT and correcting hypophosphatemia, with denosumab studied in this group but requiring the same hypocalcemia precautions noted above (14). Both phenotypes are treated more fully as special populations in Section 7.

### 6.5 Patient education, adherence and shared decision-making

Treatment fails in CKD-MBD less often because a drug does not work than because the regimen is hard to live with: intricate diets, a heavy binder pill burden, gastrointestinal side effects, difficult interdialytic control and a limited grasp of long-term risk. Recognizing high-phosphate foods and additives, timing binders to meals, and watching for the adverse effects of vitamin D and calcimimetics all take continuing education, and a patient told only to eat less phosphate, without concrete alternatives, tends to eat too little protein or to abandon the regimen. Shared decision-making matters most in anti-osteoporotic therapy, where denosumab, bisphosphonates, teriparatide and romosozumab each offer a possible reduction in fracture set against uncertainty over hypocalcemia, low-turnover disease, cost and cardiovascular risk; explaining the limits of the evidence and the monitoring plan lets the goal move from a higher bone density toward fewer fractures and preserved function. Good management, in short, joins drug therapy to nutritional education, fall prevention, an honest reckoning of pill burden, laboratory follow-up, repeat imaging and multidisciplinary care.

## 7 Individualized management in special populations

The phenotypes above cut across clinical settings, but several populations deserve specific comment. Hemodialysis patients, with little residual function, limited dialytic phosphate clearance, higher protein needs and a heavy medication burden, tend toward persistent hyperphosphatemia, a fluctuating PTH and progressing calcification. Two concerns stand out. One is the cumulative calcium load from calcium-containing binders, dialysate calcium and active vitamin D combined. The other is the low-turnover bone disease that follows over-suppression of PTH, which weakens the skeleton’s buffering of calcium and phosphate and may promote calcification, so many hemodialysis patients sit at the intersection of the high-turnover and calcification-principal management priority and must be managed for both (5, 6, 36).

Peritoneal dialysis has its own profile. Residual function usually lasts longer, so early phosphate control can be better than on hemodialysis, but as that function fades and adequacy falls short, phosphate and PTH grow harder to hold. The glucose load, protein loss, inflammation and malnutrition tied to peritoneal fluid bear on bone metabolism, and mineral clearance varies with peritoneal transport status, so the hemodialysis playbook does not transfer directly. How dialysis modality, residual function and adequacy shape fracture and calcification over the long term should be interpreted through serial, guideline-based monitoring rather than by extrapolating directly from hemodialysis (10, 42).

Transplantation does not end the story, and the post-transplant phenotype is where its distinct bone disease appears. Long-standing pre-transplant CKD-MBD, persistent parathyroid hyperplasia, glucocorticoids and immunosuppressants, hypophosphatemia, tubular phosphate loss and shifting graft function all bear on bone health, and some recipients lose bone rapidly in the early months, with vertebral and hip fracture a particular concern. Denosumab increases bone density in transplant recipients but carries the same hypocalcemia caution (14). Compared with dialysis, the emphasis shifts from high phosphate and SHPT toward preventing osteoporosis, managing persistent SHPT, correcting hypophosphatemia and guarding against immunosuppression-related bone damage, and restored kidney function should not be read as resolved CKD-MBD.

Diabetic CKD carries a more tangled bone phenotype, overlapping heavily with the low-turnover phenotype. Diabetes lowers bone quality through advanced glycation end products, microvascular disease, oxidative stress, sarcopenia and falls, so fracture risk can rise even when density does not, and low-turnover or adynamic bone disease is more common, which makes PTH harder to read (130, 131). Treating from density or PTH alone risks missing the loss of bone quality or misjudging turnover, so fracture history, fall risk, turnover markers and imaging all deserve weight (132, 133).

Older CKD patients bring age-related osteoporosis, CKD-MBD, sarcopenia, frailty, polypharmacy and cognitive decline together, and here the goal reaches past laboratory values to fewer falls, fractures, admissions and losses of independence (134). This group is more sensitive to hypocalcemia, hypercalcemia, constipation, gastrointestinal effects and drug interactions, and binder pill burden and dietary restriction weigh more heavily on daily life. The pragmatic course is to correct clearly abnormal values while protecting nutrition and function, to assess anti-osteoporotic therapy actively in those at high fracture risk, and to avoid the new harms that over-treatment can bring. As noted in Section 1.1, CKD-MBD in children raises distinct issues of growth and skeletal maturation and is not covered here.

## 8 Future directions: from surrogate endpoints toward testable precision management

The central task ahead is to turn marker control into benefit that can be tested. Abnormal phosphate, PTH, FGF23 and vitamin D correlate with poor outcomes, but correlation is not benefit, and trials should adopt fracture, cardiovascular events, hospitalization, death, quality of life and function as endpoints rather than a fall in phosphate or PTH. CKD-associated osteoporosis needs an evidence base of its own, since a population at high fracture risk is routinely excluded from general osteoporosis trials and left without safety or efficacy data (11–17, 82, 83, 115–121). Non-invasive diagnosis needs the same investment: with biopsy hard to scale and PTH alone unreliable, bone-specific alkaline phosphatase, P1NP, TRAP5b, DXA, HR-pQCT, vertebral imaging, calcium score and calcification propensity could be combined into a prospectively validated risk model (39–41, 70–77). Above all, the five-phenotype framework proposed here is a hypothesis, not a validated classification, and its value depends on whether the phenotypes can be defined reproducibly and shown to predict outcomes and to modify treatment response.

### 8.1 Evidence quality and clinical trial design

CKD-MBD has long suffered from rich mechanism and thin outcome evidence, for reasons worth separating. Clinical endpoints take time to appear, and fracture, cardiovascular events and death are shaped by age, diabetes, dialysis modality, nutrition and comedication, so a single mineral intervention seldom shows a short-term difference. The population is heterogeneous, so one PTH level can mean different turnover states in different patients, and one drug can carry opposite risk–benefit in high- versus low-turnover disease; without stratification, effects wash out, which is the practical argument for enrolling by phenotype. Ethics and safety narrow the field further, because the patients who most need treatment are also the most prone to hypocalcemia, hypercalcemia, cardiovascular events, infection and drug accumulation, which is why many osteoporosis trials excluded low eGFR or dialysis and left extrapolation shaky. And a reliance on phosphate or PTH as primary endpoints, convenient to observe, has made patient benefit hard to demonstrate, as EVOLVE showed for cinacalcet (69). The remedy is to pair short-term mechanistic endpoints with long-term clinical ones, from phosphate, PTH and bone turnover markers to BMD, vertebral and non-vertebral fracture, calcification progression and quality of life.

Better trials share a few features. They stratify at entry by CKD stage, dialysis status, PTH, turnover markers, fracture history and calcification, which is to say by phenotype. They pre-define safety endpoints such as hypocalcemia, hypercalcemia, low-turnover disease and cardiovascular events. They resist treating all CKD-MBD as one population, and instead answer specific questions: a calcimimetic strategy in dialysis patients with a high-turnover phenotype, a bone-forming approach in CKD G4-G5D patients at high fracture risk with a normal PTH, or a low-calcium-load strategy in the calcification-principal management priority. And they let real-world data and randomized trials complement each other, the cohorts carrying long-term safety and the trials carrying causality and efficacy.

### 8.2 Priority questions in mechanistic research

Several mechanistic questions stand out. The FGF23–Klotho axis needs to move from correlation to causal stratification, distinguishing FGF23 as a compensatory signal, a risk marker and a possible mediator, and testing whether preserving Klotho can improve cardiovascular and skeletal outcomes without worsening hyperphosphatemia; kidney-specific Klotho and osteocyte FGF23 models and varied phosphate loads suit the bench, while the clinic needs longitudinal analysis linking FGF23, Klotho, phosphate, PTH and cardiac structure.

Non-invasive discrimination of turnover is the bottleneck for translation. PTH and bone-specific alkaline phosphatase help but cannot replace biopsy, so the goal is a predictive model combining marker panels, imaging, machine learning and clinical features, validated externally against biopsy cohorts; a reliable separation of high from low turnover would make both anti-osteoporotic and SHPT treatment far more precise, and would give the phenotype framework an objective basis.

Vascular calcification should expand from imaging endpoints toward formation and reversibility. Most studies watch the calcium score, but the activity of calcification, crystal maturity, inflammation and plaque stability may reflect risk better than total burden, and CPPs, T50, 18F-NaF PET/CT and calcification-related proteins may flag the process while it is still active. The questions that matter are which calcification remains modifiable and which is only a scar of past injury, and which treatments cut events rather than merely shift an image.

The gut microbiota and immune inflammation need to be studied with mineral metabolism, not as separate enthusiasms. A breached gut barrier raises the toxin and inflammatory burden, and inflammation in turn drives resorption and calcification, so it is worth asking whether high-fiber diets, prebiotics, intestinal adsorbents, fewer phosphate additives and anti-inflammatory measures can improve FGF23, PTH, turnover and calcification propensity at once. Finally, patient-reported outcomes deserve more weight than they receive: bone pain, pruritus, muscle weakness, fear of falling, pill burden and dietary restriction are much of what patients live with, and a lower phosphate or PTH bought with more pills and more dietary anxiety is not obviously a gain.

## 9 Conclusion

CKD-MBD is where the systemic reach of CKD becomes visible, and its significance runs well past any single value of calcium, phosphate, PTH or vitamin D. Understanding has widened from disordered mineral metabolism to a multi-organ syndrome that draws in the kidney, skeleton, vasculature, heart, immune system and gut. Three tensions still define management: biochemistry is easy to measure but a poor proxy for fracture and cardiovascular risk; biopsy is accurate but not scalable; and the available drugs move surrogate markers while the evidence for hard endpoints stays limited, as the EVOLVE result illustrates.

The way forward proposed here is to treat CKD-MBD as bone–vascular risk medicine: to read the syndrome through a small set of recurring phenotypes, to grade honestly the evidence behind each mechanism and intervention, and to judge treatment by patient outcomes rather than by the numbers it normalizes. The five-phenotype framework is offered as a hypothesis to be tested, not a finished classification. Whether or not it survives validation, the underlying shift it embodies, from correcting a number toward stratifying risk and improving endpoints, is what will let the field deliver what patients with CKD actually need: fewer fractures, better-protected hearts and vessels, and a better quality of life.
